# BSN723T Prevents Atherosclerosis and Weight Gain in ApoE Knockout Mice Fed a Western Diet

**Published:** 2015-12-12

**Authors:** Jarrod Williams, Charles Ensor, Scott Gardner, Rebecca Smith, Robert Lodder

**Affiliations:** University of Kentucky, Department of Pharmaceutical Sciences - United States of America; University of Kentucky, Department of Pharmaceutical Sciences - United States of America; University of Bath - United Kingdom; University of Kentucky, Department of Pharmaceutical Sciences - United States of America; Pharmaceutical Sciences, BPC223 Biopharmaceutical Complex, 40536 - United States of America

**Keywords:** D-tagatose, D-lyxohexulose, dihydromyricetin, DLH, atheroma, sucrose, fat, ApoE-/-

## Abstract

**Objective:**

This study tests the hypothesis that BSN723T can prevent the development of hyperlipidemia and atherosclerosis in ApoE^-/-^ knockout mice fed a Western (high fat, high cholesterol, and high sucrose) diet. BSN723T is a combination drug therapy consisting of D-tagatose and dihydromyricetin (BSN723).

**Background:**

D-tagatose has an antihyperglycemic effect in animal and human studies and shows promise as a treatment for type 2 diabetes and obesity. Many claims regarding BSN723's pharmacological activities have been made including anti-cancer, anti-diabetic, anti-hypertensive, anti-inflammatory, and anti-atherosclerotic effects. To our knowledge this is the first study that combines D-tagatose and BSN723 for the treatment of hyperlipidemia and the prevention of atherosclerosis.

**Methods:**

ApoE-deficient mice were randomized into five groups with equivalent mean body weights. The mice were given the following diets for 8 weeks: Group 1 - Standard diet; Group 2 - Western diet; Group 3 - Western diet formulated with D-tagatose; Group 4 - Western diet formulated with BSN723; Group 5 - Western diet formulated with BSN723T. Mice were measured for weight gain, tissue and organ weights, total serum cholesterol and triglycerides and formation of atherosclerosis.

**Results:**

The addition of D-tagatose, either alone or in combination with BSN723, prevented the increase in adipose tissue and weight gain brought on by the Western diet. Both D-tagatose and BSN723 alone reduced total cholesterol and the formation of atherosclerosis in the aorta compared to mice on the Western diet. Addition of BSN723 to D-tagatose (BSN723T) did not increase efficacy in prevention of increases in cholesterol or atherosclerosis compared to D-tagatose alone.

**Conclusion:**

Addition of either D-tagatose or BSN723 alone to a Western diet prevented weight gain, increases in total serum cholesterol and triglycerides, and the formation of atherosclerosis. However, there was no additive or synergistic effect on the measured parameters with the combination BSN723T treatment.

## Introduction

Evidence strongly supports a link between obesity and a spectrum of diseases including type 2 diabetes, hypertension, hyperlipidemia, and cardiovascular disease. Hyperlipidemia is commonly seen in those who are obese and those with type 2 diabetes and is thought to be a major contributor to the increased incidence of cardiovascular disease seen in these populations^1,2^. Such secondary hyperlipidemia is typically characterized by elevated levels of triglycerides and low-density lipoprotein (LDL) cholesterol and by low levels of high-density lipoprotein (HDL) cholesterol. Reduction of elevated LDL and raising of HDL have been major drug treatment goals, and drugs have been developed that alter blood lipids and produce significant reduction in cardiovascular events in patients with cardiovascular disease and diabetes^3,4^.

Elevated serum cholesterol levels have been noted in rodents^5^, dogs^6^, nonhuman primates^7^, and humans^8^ consuming a high-carbohydrate diet, particularly one including fructose and sucrose. Studies have provided evidence that fructose causes hypertriacylglycerolemia postprandially both directly through decreased triglyceride clearance, and indirectly by increasing liver re-esterification of fatty acids^9^. Low-density lipoprotein receptor deficient (LDLr^−/−^) mice fed a high sucrose diet exhibited elevated serum LDL cholesterol concentrations and increased atherosclerosis compared to mice fed an energy-matched diet enriched in saturated fatty acids^10^.

D-tagatose, a naturally occurring epimer of fructose, was originally developed as a low-calorie sweetener (1.5 kcal/g compared to 4 kcal/g for sucrose) but was found to have an antihyperglycemic effect in animal and human studies and shows promise as a treatment for type 2 diabetes and obesity^11-14^. After more than 10 years of animal and human studies, D-tagatose was classified as being “generally recognized as safe (GRAS)” by the FDA and has been used since in food and beverage products with no serious adverse events reported^15^.

The mechanism by which D-tagatose produces its antihyperglycemic effect in response to a meal is not clear. Based on studies with fructose and D-tagatose, it was proposed that D-tagatose is metabolized following a pathway that is essentially the same as that of fructose^15^. After absorption from the intestine and transport to the liver, fructokinase phosphorylates D-tagatose to produce D-tagatose-1-phosphate. D-tagatose-1-phosphate can stimulate glucokinase activity^16-17^ leading to increased phosphorylation of glucose to glucose-6-phosphate and further activating glycogen synthase^18^. It has been suggested that D-tagatose-1-phosphate can inhibit glycogen phosphorylase in the same manner that fructose-1-phosphate does^15^, but this has not been directly shown. By activating glycogen synthase and possibly inhibiting glycogen phosphorylase, D-tagatose-1-phosphate increases glycogen synthesis and inhibits glycogen utilization, at least partly explaining the antihyperglycemic effect of the sugar. In addition to the effect on glycogen regulation, D-tagatose inhibits sucrase^19^, leading to the suppression of sucrose digestion in the small intestine and inhibits the activity of maltase, at least in vitro, which could slow the digestion of starch. The net effect of the regulation of these enzymes is an increase in glycogen synthesis and storage and a decrease in glycogen utilization. In addition, D-tagatose reduces the absorption and digestion of sucrose and other carbohydrates in the small intestine. D-tagatose has been shown in both animal and human studies to have multiple effects including increase in satiety and weight control, a beneficial effect on abnormal blood lipids, a reduction in atherosclerotic plaque formation, and a reduction in blood glucose and HbA1c levels in patients with type 2 diabetes mellitus ^11,13-15,20-26^.

In addition to its antihyperglycemic effects, D-tagatose has been found to have an effect on blood lipid levels in animals and in humans. In one study, LDL^-/-^ mice fed a diet in which high sucrose content was replaced with an equivalent amount of D-tagatose exhibited reduced cholesterol, triglycerides, and atherosclerosis compared to mice on the diet containing sucrose^26^. In a human clinical trial, patients with type 2 diabetes taking D-tagatose were found to have improved HDL levels, increasing from 30 to 41.7 mg/dL over the course of the 14 month study^22^. This is interesting in light of evidence suggesting that increasing HDL levels decrease the risk of incurring a coronary event. The mechanism by which D-tagatose raises HDL is not clear, but it should be noted that these patients did lose weight during the study and this may have contributed to the improvement in HDL. In other studies, type II diabetics taking D-tagatose showed a decrease in HbA1c and serum triglycerides^11-12^.

Dihydromyricetin (BSN723 or DMY), also known as ampelopsin, is a flavonoid that has been isolated from a number of plants, including *Ampelopsis grossedentata, Cedrus deodara*, *Hovenia dulcis*, and *Erythrophleum africanum*, that have been used in traditional medicine. Many claims have been made regarding dihydromyricetin's numerous health benefits including antioxidant properties^27^, anti-cancer^28-30^, anti-hypertensive^31^, anti-inflammatory^32^, and anti-atherosclerotic effects^33^. Dihydromyricetin is also indicated as a treatment for alcohol intoxication^34^ and a preliminary study suggests it as a possible treatment for Alzheimer's disease^35^.

A number of published studies provide evidence that dihydromyricetin can protect cells against oxidative injury (for example, see Zhang et al. 2003^27^, Ye et al. 2008^36^, Lin et al. 2014^37^, Zou et al. 2014^38^). Recently, Jiang et al. (2014)^39^ examined the effects of dihydromyricetin on oxidative stress and glucose transport activity in a methylglyoxal (MG)-induced PC12 cell line to explore the possibility of using dihydromyricetin for the treatment of MG-induced diabetes-associated cognitive decline. They found that DMY protected PC12 cells against MG-induced apoptosis and glycometabolic disorders, at least in part by restraining the hyperactivation of p-AMPK activity and normalizing the translocation of GLUT4 from the intracellular compartment, resulting in a balance in glucose uptake.

Much attention has been focused on the use of dihydromyricetin in the treatment of a variety of cancers and there have been many studies, both in vitro and in vivo, demonstrating inhibitory activity of dihydromyricetin against cell lines of breast cancer^40^, liver cancer ^29-30,37^, melanoma^41^, osteosarcoma^42^, and lung cancer^43-44^. Dihydromyricetin has also shown anticancer activity against bladder cancer^45^, lung cancer^46^, and prostate cancer^47^ xenografts, and showed synergistic effects with adriamycin for treating leukemia xenografts^48^.

Recently, Chen et al. (2015)^49^ looked at the effects of dihydromyricetin on nonalcoholic fatty liver disease (NAFLD) in a clinical study. The pathogenesis of NAFLD includes insulin resistance, oxidative stress, mitochondrial dysfunction and inflammation in the liver.

The study looked at inflammatory mediators and biomarkers of NAFLD as well as glucose and lipid metabolism. They found that while dihydromyricetin did not alter the severity of fatty infiltration in the liver, it did produce significant improvements in several liver enzymes and reduced serum levels of several markers including tumor necrosis factor-alpha, cytokeratin-18, and fibroblast growth factor 21. They also found that the HOM-IR level was decreased in dihydromyricetin treated patients, but insulin and C-peptide levels were not affected. Levels of low-density lipoprotein-cholesterol (LDL-C) and apolipoprotein B (Apo B) were also significantly decreased by dihydromyricetin, but the total cholesterol, triglyceride, high-density lipoprotein-cholesterol (HDL-C), and Apo-A-I concentrations did not significantly differ between treated and control groups. Other evidence that dihydromyricetin can affect glucose metabolism include studies that found that DMY activated insulin signaling and increased glucose uptake in skeletal muscle in vitro and in vivo ^50,51^.

Recent studies have begun to offer clues about dihydromyricetin's mechanism of action. It is now known that a number of cell signaling pathways are affected by dihydromyricetin. Zou, et al. (2014)^38^ found that dihydromyricetin fed to rats for 7 days increased the expression of peroxisome proliferator-activated receptor γ coactivator 1α (PGC-1α) in skeletal muscle. PGC-1α is known to regulate irisin, an exercise-induced myokine that can stimulate the browning of white adipose tissue. In a follow-up study (Zhou, et al., 2015)^52^ the effect of dihydromyricetin on irisin secretion through the PGC-1α pathway was investigated in vivo (in rats and humans) and in vitro (L6 myotubes). The results were an increase in irisin secretion with the administration of dihydromyricetin.

Dihydromyricetin has also been found to increase the levels of phosphorylated AMP activated protein kinase (AMPK) and Ulk1, and decrease phosphorylated mTOR levels^50^. The same group also found that dihydromyricetin increased levels of peroxisome proliferator-activated receptor coactivator-1α (PGC-1α), and Sirt3 in skeletal muscle in vitro and in vivo^51^. Jiang, et al. (2014) found that dihydromyricetin ameliorates the oxidative stress response induced by methylglyoxal via the AMPK/GLUT4 signaling pathway^39^.

A previous study combining D-tagatose with another naturally occurring antioxidant, polydatin, found the combination to be effective in lowering total cholesterol and preventing the formation of atherosclerosis in ApoE^-/-^ mice^53^. There has been relatively little published research regarding DMY combination drugs or dihyromyricetin's effect on cardiovascular health. Chen et al. (2015)^49^ recently reported that dihydromyricetin lowered LDL cholesterol in a human clinical trial. A second study reported that administering an extract from *A. grossedentata* to rats reduced serum total cholesterol and triglycerides and increased high-density lipoprotein, and that humans given a drink made from *A. grossedentata* showed a reduction in serum triglycerides, total cholesterol, and plasma lipid ^33^.

In the present study, we aim to examine the effect of BSN723T, a combination of D-tagatose and dihydromyricetin, on blood lipids and atherosclerosis in ApoE^-/-^ knockout mice. The apoE lipoprotein resides on very low, intermediate, and high density lipoproteins (VLDL, IDL, and HDL, respectively) and mediates the removal of lipoproteins from plasma by acting as a ligand for low density lipoprotein (LDL) receptors. Mice are normally resistant to the development of atherosclerosis, however inactivation of the apoE gene in mice results in elevated cholesterol and triglycerides in these mice. Severe hypercholesterolemia and the rapid development of atherosclerosis results when these mice are fed a Western type diet consisting of high fat, high cholesterol and high sucrose^54-57^. The progression and histopathology of lesions in this animal model show features similar to those observed in humans and other species, making these mice good models for evaluating diet composition and potential drugs for their effect on atherosclerotic development^58^. We tested the hypothesis that BSN723T lowers serum triglycerides and cholesterol, and prevents the formation of atherosclerosis in apoE^-/-^ mice fed a Western diet. This is the first known study examining the combined effects of D-tagatose and BSN723 on blood lipids and the development of atherosclerosis.

## Methods

### Materials

D-tagatose was obtained from Inalco, S.p.A. (Milano, Italy). Dihydromyricetin [(2R,3R)-3,5,7-trihydroxy-2-(3,4,5--trihydroxyphenyl)- 2,3-dihydro--chromen-4-one] was obtained from Qingdao Ai Weisheng Chemical Co. Ltd. (Shandong, China).

### Mice and Diets

The animal-use protocol was approved by the University of Kentucky Institutional Animal Care and Use Committee. Male ApoE^-/-^ mice (C57BL/6 background) 14 to 18 weeks of age were obtained from Taconic Biosciences. The mice were given water *ad libitum* and kept on a 12 hour light/dark cycle. Mice were randomized into 5 groups (n = 10 per group) to produce groups with the equivalent mean body weights.

Group 1 (Standard diet) is a negative control group for atherosclerosis and was fed Standard chow (TD.2018, see Illustration 1 for diet compositions) for the duration of the study.

Group 2 (Western diet) is a positive control group for atherosclerosis and was fed Standard chow for the two week D-tagatose run-in period (see below) and then switched to a high fat, high cholesterol, high sucrose Western diet (TD.88137) for the remainder of the study.

Group 3 (D-tagatose diet) was on Standard chow during the two week run-in period during which the mice of this group were given gradually increasing concentrations of D-tagatose in the water every day until the total daily mass of D-tagatose drank by the mice matched the mass consumed in the study chow, assuming average daily consumption of water and chow (see Illustration 2). These mice were then switched to a Western diet chow (TD.140143), in which the sucrose was replaced by D-tagatose, for the remainder of the study.

Group 4 (BSN723 diet) was on Standard chow during the two week run-in period and then switched to a Western diet (TD.140144) with dihydromyricetin (BSN723) added and kept on this diet for the remainder of the study.

Group 5 (BSN723T diet) was on Standard chow during the two week run-in period during which the mice of this group were given gradually increasing concentrations of D-tagatose in the water every day until the total daily mass of D-tagatose drank by the mice matched the mass consumed in the study chow (see Illustration 2), assuming average daily consumption of water and chow. These mice were then switched to normal water and a Western diet (TD.140145), in which the sucrose was replaced by D-tagatose and with dihydromyricetin added, for the remainder of the study.

TD.140143, TD.140144, and TD.140145 were custom formulations by Harlan Teklad.

### Study Design

An outline of the time course of the study is shown in Illustration 3.

Due to the potential for gastrointestinal distress that results from the poor absorption of D-tagatose, ApoE^-/-^ mice receiving D-tagatose in their diets (Groups 3 and 5) were acclimated to the sugar by adding increasing amounts of D-tagatose to their drinking water daily during a two week run-in phase before beginning their respective diets (Illustration 2). Tagatose was not placed in the chow for the tagatose run-in period because previous experience with powdered chow feeders had shown that much of the chow was lost in the litter and it was too difficult to weigh food consumption. Additionally, because there is a minimum order for custom chows, it would not have been cost effective to make pelleted chow for all of the different tagatose doses used in the run-in period.

After the D-tagatose run-in period, mice were maintained on their diets for 8 weeks. Compositions of the diets are shown in Illustration 1. Most of the carbohydrate content of the Western diet comes from sucrose. In contrast, while having the highest carbohydrate caloric content, the carbohydrate in the Standard chow is from complex carbohydrates found in grain components, there is no sucrose added. In the TD.140143 and TD.140145 chows, the sucrose has been replaced by D-tagatose. The ratios of calories from fat, protein and carbohydrates (fat / protein / carbohydrates) are as follows: TD.2108 (Standard) =18/24/58; TD.88137 (Western) = 42/15/43; TD.140143 (D-tagatose) = 52/19/29; TD.140144 (BSN723) = 42/15/43; TD.140145 (D-tagatose + BSN723) = 52/19/29.

Body weight and food consumption was measured during the D-tagatose run-in phase and weekly thereafter during the dosing phase. Blood was sampled by submandibular bleeds after overnight fasts at multiple time points throughout the treatment period (see Illustration 3), and total serum cholesterol and triglyceride levels were determined.

At the study end point, mice were euthanized using CO_2_ followed by cervical dislocation, and the left ventricle was punctured to obtain blood. After the right atrium was cut, mice were exsanguinated by perfusion with saline through the left ventricle and tissues (liver, kidneys, spleen, epididymal fat, retroperitoneal fat, subcutaneous fat and aortic tissue from the heart to iliac bifurcation) were removed. The livers, kidneys, spleens and fat tissues were weighed. The hearts with the aortas attached were fixed overnight in 4% paraformaldehyde made with phosphate buffered solution, and then transferred to phosphate buffered solution for storage.

### Blood analysis

Total serum cholesterol and total serum triglyceride levels were determined using enzymatic assay kits (Wako Pure Chemical, Richmond, VA).

### Atherosclerosis measurements

Aortas were prepared for atherosclerosis measurements via en face presentation, in which the entire length of the aorta was removed from the animal, the entire intimal surface and greater curvature of the aortic arch exposed, and the resulting tissue pinned to a dark surface. For determining the area of the aortic arch, a 3 mm line was drawn in software downward from the root of the left subclavian artery. Using the bottom of this line as a base, the intimal area of the arch was traced to determine the area. The atherosclerotic plaques were then traced and quantified using Nikon NIS Elements software^26^. Atherosclerotic lesions were quantified by two independent observers (SG and JW). Data are expressed as the percentage of the aortic arch covered with grossly discernable atherosclerotic lesions.

### Calculations and Statistics

Data are presented as the mean ± standard error of the mean (s.e.m.). 1-way ANOVA was utilized for analyses. Values of *P* < 0.05 were considered to be statistically significant.

## Results

One mouse in Group 1 died on day 42 after being bled. One mouse in Group 4 died on day 44 and was found on necropsy to have had encephalitis and meningitis of unknown origin. Data gathered from these two mice up until the time of their deaths were included in the analysis.

### Food Consumption

Based upon the weight of food eaten by each group of mice and the number of Kcal/g available for each diet, average daily energy consumption per mouse was calculated (Illustration 4). The mice on the Western and BSN723 diets consumed the highest number of calories per day (15.77 ± 1.13 Kcal/day and 16.34 ± 0.98 Kcal/day, respectively, no significant difference, *P* = 0.69) followed by the BSN723T (13.86 ± 0.98 Kcal/day), D-tagatose (11.65 ± 1.33 Kcal/day) and Standard diet (11.23 ± 0.49 Kcal/day) groups. The caloric intake between the following groups was statistically significant; Standard and Western diets (**+** *P* < 0.001), Standard and BSN723 diet (**+** *P* < 0.001), Standard and BSN723T diet (**+** *P* = 0.004), Western and D-tagatose diet (* *P* < 0.001), D-tagatose and BSN723 diet (* *P* < 0.001) and the BSN723 and BSN723T diet groups (◆ *P* = 0.014). There was no significant difference in caloric intake between the Standard and D-tagatose, Western and BSN723, Western and BSN723T or D-tagatose and BSN723T diet groups. There was no significant difference between the five groups in terms of food consumption by weight of chow eaten. One possible caveat regarding the estimated caloric values assigned to the chows containing D-tagatose is that the exact caloric value of the sugar is not a certainty and probably varies depending on the individual consuming it. Upon initial consumption of the sugar, it is poorly absorbed by the intestine, but absorption may increase over time with continued consumption. A value of 1.5 kcal/g of D-tagatose has been agreed upon for the use on food labels by the FDA and this is the value used to calculate the number of kcal/g in the chows containing D-tagatose^59^. Oxygen consumption measurements for each mouse in each study may be the best way to determine energy consumption in a particular study, but given the dependence of tagatose caloric content on the gut microbiome, such results may not be transferrable to other studies.

### Body Weights

Mice in all five groups were on the Standard diet at the beginning of the study and during the two week D-tagatose run-in period. At the end of the run-in period the body weights of the mice in all five groups were not significantly different (Illustration 5A). On day 15 the mice were placed on their respective diets for 8 weeks.

There was a drop in weight in the D-tagatose and BSN272T groups (Groups 3 and 5) between day 15, when the mice were started on their respective D-tagatose containing chows, and day 21 when the mice were next weighed. The mice in these two groups then gained weight at a rate comparable to the Standard diet mice and at the end of the study there was no significant difference between the weights of the mice in these three groups. Food consumption by mice in the D-tagatose and BSN272T groups was decreased compared to the other three groups during the first week the mice were placed on their respective diets and this is the likely explanation for the weight loss. After the first week, their food consumption increased and there was no significant difference between the five groups in terms of food consumption by weight of chow eaten. At the end of the study, mice on the Western diet weighed the most (37.5 ± 1.46 g) but not significantly more than mice on the BSN723 diet (34.6 ± 1.04 g, *P* = 0.13). Mice on the Western and BSN723 diets weighed significantly more (* *P* ≤ 0.024) than mice on the other three diets while there was no significant difference between the weights of mice on those three diets (Standard diet, 31.2 ± 0.89 g; D-tagatose diet, 29.5 ± 1.40 g; BSN723T diet, 29.2 ± 0.83 g). Differences in caloric intake between the 5 groups directly correlates with the final body weights of each group of mice and could account for any differences between the groups (Illustration 5B).

### Adipose tissue and organ weights

Mice on the Western diet had increased total adipose tissue (epidydimal + retroperitoneal + subcutaneous adipose tissue) compared to mice on the Standard diet (Illustration 6). The addition of D-tagatose, either alone or in combination with BSN723, prevented the increase in adipose tissue brought on by the Western diet and, in fact, mice consuming D-tagatose (Groups 3 and 5) were leaner than the mice on the Standard diet. Mice on the BSN723 diet were also leaner than those on the Western diet but were not significantly different from mice on the Standard diet. While mice on the BSN723 had more epididymal fat than mice on the Standard diet (0.67 ± 0.14 g vs 0.47 ± 0.09 g, respectively), the difference was not statistically significant (*P* = 0.26). The amount of subcutaneous (0.2 ± 0.03 g) and retroperitoneal fat (0.23 ± 0.06 g) in mice on the BSN723 mice was similar to that found in mice on the Standard diet (0.19 ± 0.04 g and 0.29 ± 0.07 g, respectively).

Livers from mice on the Western diet weighed significantly more than livers from mice on all the other diets (*P* < 0.02) (Illustration 7A). There was no difference between livers of mice from any of the other groups. The only significant differences in the weights of spleens were from mice on the Western and BSN723 diets compared to spleens from mice on the Standard diet (*P* < 0.02) (Illustration 7B). There was no difference between spleens of mice from any of the other groups. The lungs from mice on the D-tagatose diet (Group 3) were slightly smaller (*P* = 0.012) than the lungs from the Standard diet fed mice (Illustration 7C). There were no differences between any of the other groups. Kidneys from mice that received D-tagatose in their diets (groups 3 and 5) were slightly smaller than kidneys from mice in the other groups (*P* < 0.02) (Illustration 7D).

During the D-tagatose 14 day run-in period all of the mice were on the Standard diet (day 1 to 14). At the end of the 14 day run-in period, there were no significant differences in serum total cholesterol levels between any of the groups (Illustration 8). On day 15, the mice were started on their respective diets. By day 36, cholesterol had increased in the mice on the Western diet and the three treatment diets (Groups 3, 4 and 5) compared to mice on the Standard diet. However, the increase was significantly less in the two groups receiving D-tagatose (* Group 3 and 5, *P* < 0.0001 for both groups) compared to mice on the Western diet. There was no significant difference in cholesterol between the mice on the Western and BSN723 diets on day 36. By Day 71 (end of study) total cholesterol in all three treatment groups was significantly less (^+^ D-tagatose, *P* = 0.034; BSN723, *P* = 0.005; and BSN723T, *P* = 0.016) than that of mice on the Western diet, but significantly higher than mice on the Standard diet (^+^ D-tagatose, *P* = 0.0006; BSN723, *P* = 0.012; and BSN723T, *P* = 0.0007).

### BSN723 and D-tagatose lower serum triglycerides compared to mice on both the Standard and Western diets

At the end of the 14 day run-in period, there were no significant differences in triglycerides between any of the groups (Illustration 9). There was also no significant difference in triglyceride levels in mice on the Standard or Western diets at any time point during the study. The D-tagatose, BSN723, and BSN723T diets lowered triglycerides compared to mice on the Standard and Western diets during the course of the study. By day 71, triglycerides in Groups 3, 4, and 5 were approximately half or better than the levels in either the Standard or Western diet groups (Standard diet, 263.7 ± 33.1 mg/dL; Western diet, 256.0 ± 31.9 mg/dL; D-tagatose, 102.9 ± 5.65 mg/dL; BSN723, 155.0 ± 15.1 mg/dL; BSN723T, 124.7 ± 12.3 mg/dL; *P* ≤ 0.014 Groups 3, 4, and 5 compared to Groups 1 and 2.) On Day 71, triglycerides were also significantly lower in the D-tagatose group than in the BSN723 group (P = 0.009).

### Atherosclerosis

The surface area of aortas covered by atherosclerotic lesion was greater in mice on the Western diet compared to mice on the Standard diet (*P* = 0.001) (Illustration 10). The addition of D-tagatose, BSN723, or BSB723T to the diets inhibited the formation of plaque in aortas compared to mice on the Western diet (*P* = 0.007, *P* = 0.04, *P* = 0.016, respectively). The aortas from mice that were on the D-tagatose, BSN723, and BSN723T diets all had greater plaque formation compared to mice on the Standard diet (*P* = 0.023, *P* < 0.0001, *P* = 0.02, respectively). Significant differences were found between mice from Group 1 (Standard diet) and all other groups (*P* < 0.022), between Group 2 (Western diet) and all other groups (*P* < 0.04), and between Groups 3 (D-tagatose diet) and 4 (BSN723 diet) (*P* = 0.014).

## Discussion

Flavonoids are derivatives of 2-phenyl-1-benzopyran-4-1 and are present in fruits, vegetables, nuts, and seeds. Many studies have found an association between flavonoid intake and a reduction of risk for coronary events^60-62^. The flavonoid dihydromyricetin is the major bioactive compound in *Ampelsis grossedentata*, making up 15%–20% (wt/wt) of the total dry weight of stems and leaves^63^. Many of the preliminary studies touting the positive effects of dihydromyricetin have utilized plant extracts which, in addition to containing high concentrations of dihydromyricetin, also contained complex mixtures of other flavonoids. Chen et al.^33^ reported that the intragastric administration of extract from *A. grossedentata* to rats reduced serum total cholesterol and triglycerides and increased high-density lipoprotein. In a study using human subjects with hyperlipidemia, the administration of a drink made from *A. grossedentata* for 45 days reduced serum triglycerides, total cholesterol, and plasma lipids^33^. Up to now few studies have evaluated the serum lipid altering and anti-atherosclerotic activities of purified dihydromyricetin.

Considerable attention has been focused on the antioxidant activity of dihydromyricetin^64-65^. Liao et al.^65^ crystallized dihydromyricetin from *A. grossedentata* and demonstrated it was effective at inhibiting the production of reactive oxygen species in treated cells thereby attenuating plasma lipid peroxidation. It also inhibited AAPH-induced production of malondialdehyde, an indicator of lipid peroxidation and a biomarker for oxidative stress. Evidence suggests that oxidative stress can lead to endothelial dysfunction and is involved in the development of atherosclerotic plaque^66^.

To our knowledge this is the first study that combines D-tagatose and BSN723 for the treatment of hyperlipidemia and the prevention of atherosclerosis. This study examined the effect of the addition of BSN723, D-tagatose, or a combination of the two, on weight gain, blood lipids, and the development of atherosclerosis, in ApoE^-/-^ mice consuming a Western diet (high fat, high cholesterol, high sucrose diet). In the diets containing D-tagatose, the D-tagatose replaced the sucrose in the Western diet. For the mice treated with BSN723 alone (Group 4), sucrose was still present in the diet, BSN723 was just added to the chow formulation. The Western diet promoted obesity, increased total serum cholesterol, and markedly stimulated the development of atherosclerosis in the ApoE^−/−^ mice compared to mice on Standard chow. In contrast, a diet that replaced gram-for-gram the sucrose in the Western diet with D-tagatose, with or without BSN723, prevented the weight gain seen with animals on the Western diet and had a marked effect on blood lipids and the development of atherosclerosis.

The mice on the D-tagatose diets did not exhibit a gain in body weight witnessed in the Western diet group and weighed slightly, but not significantly, less than the Standard diet mice. A previous study also showed that replacing sucrose with D-tagatose in a Western diet fed to LDLr^-/-^ mice prevented the development of obesity^26^. We obtained the same result with ApoE^-/-^ mice. Differences in caloric intake between the 5 groups directly correlates with the final body weights of each group of mice and could account for any differences between the groups. While not significantly different, mice on the diet containing BSN723 weighed less than mice on the Western diet (34.6 ± 1.04 g compared to 37.5 ± 1.46 g, respectively) even though the caloric intake of the BSN723 group (16.34 ± 0.98 Kcal/mouse/day) was slightly, but not significantly, greater than mice on the Western diet (15.77 ± 1.13 Kcal/mouse/day).

There was a significant (*P* < 0.01) drop in weight in the two groups (Groups 3 and 5) on the diets containing D-tagatose between day 15, when the mice were started on their respective diets and day 21 when they were next weighed. The mice then gained weight at a rate comparable to the Standard diet mice and at the end of the study there was no significant difference in the weight of the mice in the two groups on D-tagatose and the group on the Standard diet. D-tagatose is known to cause gastrointestinal upset when taken in large enough doses without a gradual increase in intake, which was the reason for the 14 day run-in period. While there were no outward symptoms of gastrointestinal distress in the mice placed on the D-tagatose containing diets, they did eat less during the first week. After the first week, their food consumption increased and was comparable to the other two groups.

Interestingly, while there was no significant difference in the body weights of the mice on the Standard diet and the two D-tagatose containing diet groups (Groups 3 and 5), the mice on D-tagatose were leaner, as evidenced by the significantly lower amount of epididymal, retroperitoneal and subcutaneous fat compared to the Standard diet group. Similar results were seen in another study using LDLr^-/-^ mice^26^. The addition of BSN723 to the Western diet (Group 4) resulted in mice that were leaner than those on the Western diet, even though the caloric intake of these two groups was essentially the same, but not as lean as mice on the Standard diet or the two D-tagatose containing diets.

While D-tagatose fed mice (Groups 3 and 5) exhibited increased serum cholesterol compared to Standard diet mice, the extent of these changes was far less than those observed in the mice on the Western diet (Illustration 8). The livers of ApoE^-/-^ mice are unable to remove circulating cholesterol efficiently and as a result these mice exhibit elevated serum cholesterol. Atherosclerotic lesion development is very dramatic in ApoE^-/-^ mice fed a Western-type diet and the beginning stages of the disease can be found at 6 weeks^54^. With the ApoE^-/-^ and LDLr^-/-^ mouse models, dietary cholesterol, rather than the level of fat, exerts a major influence on the development of atherosclerosis^67-69^. In addition to cholesterol, the fatty acid profile and even the carbohydrate form (i.e. fructose, sucrose) can be manipulated to modify the atherosclerosis phenotype^10,68,70^. Replacement of sucrose with D-tagatose in a Western diet was shown to decrease serum total cholesterol in LDLr^-/-^ mice compared to mice on the Western diet with sucrose^26^. We obtained similar results with the ApoE^-/-^ mice. Total cholesterol increased somewhat in the D-tagatose, BSN723, and BSN723T fed mice, upon placing them on their respective diets compared to Standard diet, but never reached the levels of the mice on the Western diet. In future work the effect of BSN723 on HDL and LDL cholesterol should be measured and compared to D-tagatose.

In ApoE^-/-^ mice, there is no significant change in triglyceride levels when mice are placed on a Western diet compared to mice fed a Standard diet (Illustration 9). Serum triglyceride levels in ApoE^-/-^ mice are elevated compared to wild-type mice, even when fed the Standard diet. The addition of D-tagatose, BSN723, or a combination of the two, lowered serum triglycerides in these mice back toward what would be considered normal levels (see negative control). D-tagatose alone appeared to be more effective at lowering triglycerides than BSN723 alone or the BSN723T combination.

The ApoE^-/-^ mice in this study developed little atherosclerosis when maintained on the Standard diet, which does not contain sucrose or added cholesterol, and is lower in fat compared to the Western diet. All three modified diets, D-tagatose, BSN723, and BSN723T, reduced the amount of plaque formed in the aortas compared to mice on the Western diet (Illustration 10).

One of the objectives of this study was to determine if the addition of BSN723 to the diet containing D-tagatose would have any additive or synergistic effect on blood lipids or atherosclerotic plaque formation. Addition of BSN723 to D-tagatose did not increase the efficacy for reducing cholesterol or atherosclerosis compared to D-tagatose alone. While no such effect was observed, BSN723 by itself did significantly prevent diet induced rises in blood cholesterol and the formation of atherosclerosis, with efficacy increasing over time in the latter part of the study. A longer study seems warranted. Administration of BSN723 with polydatin,which yielded a synergistic reduction of lipids with D-tagatose^53^, might also prove effective.

## Figures and Tables

**Illustration 1:**
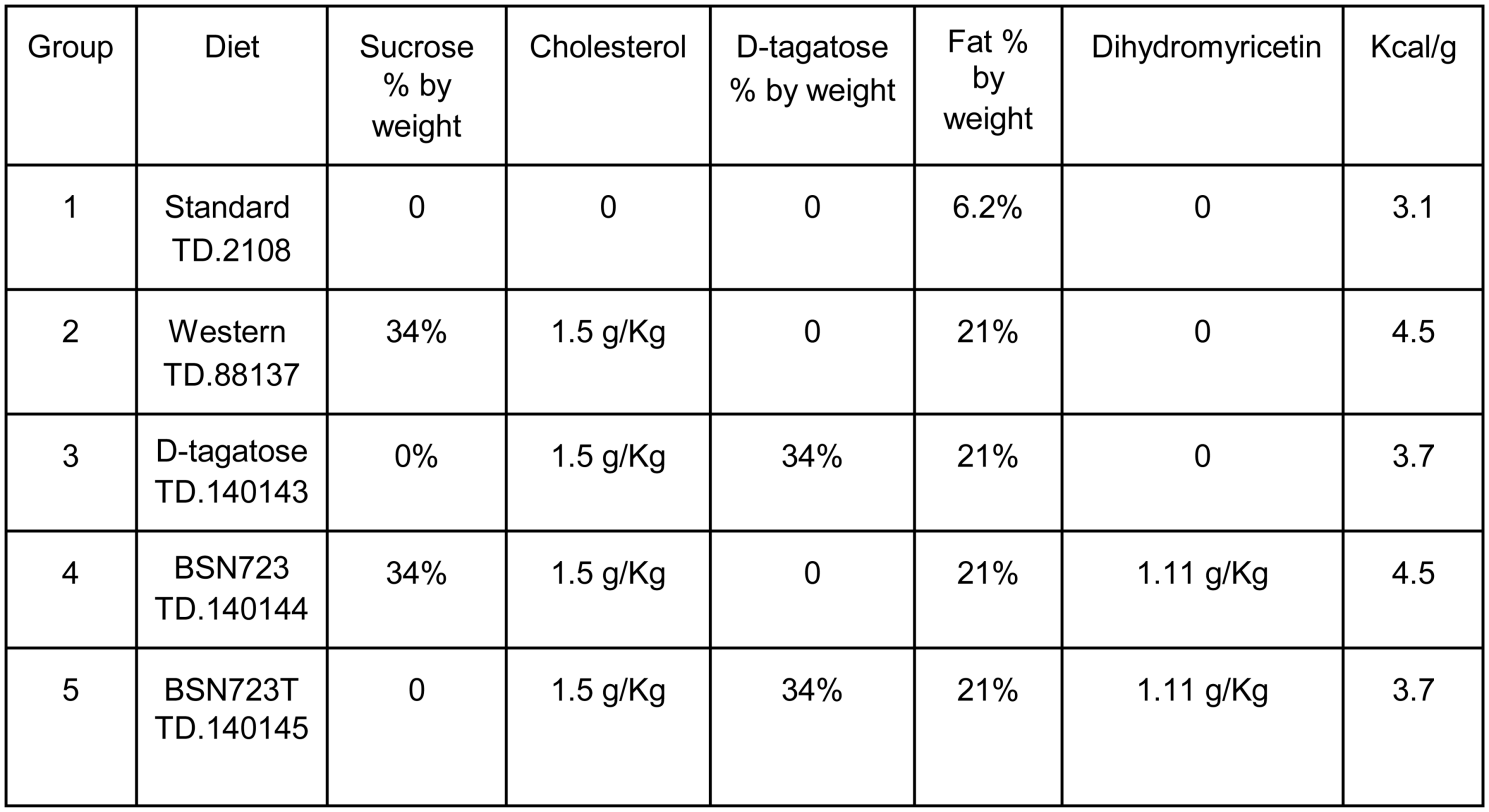
Comparison of the five diets fed to ApoE-/- mice.

**Illustration 2:**
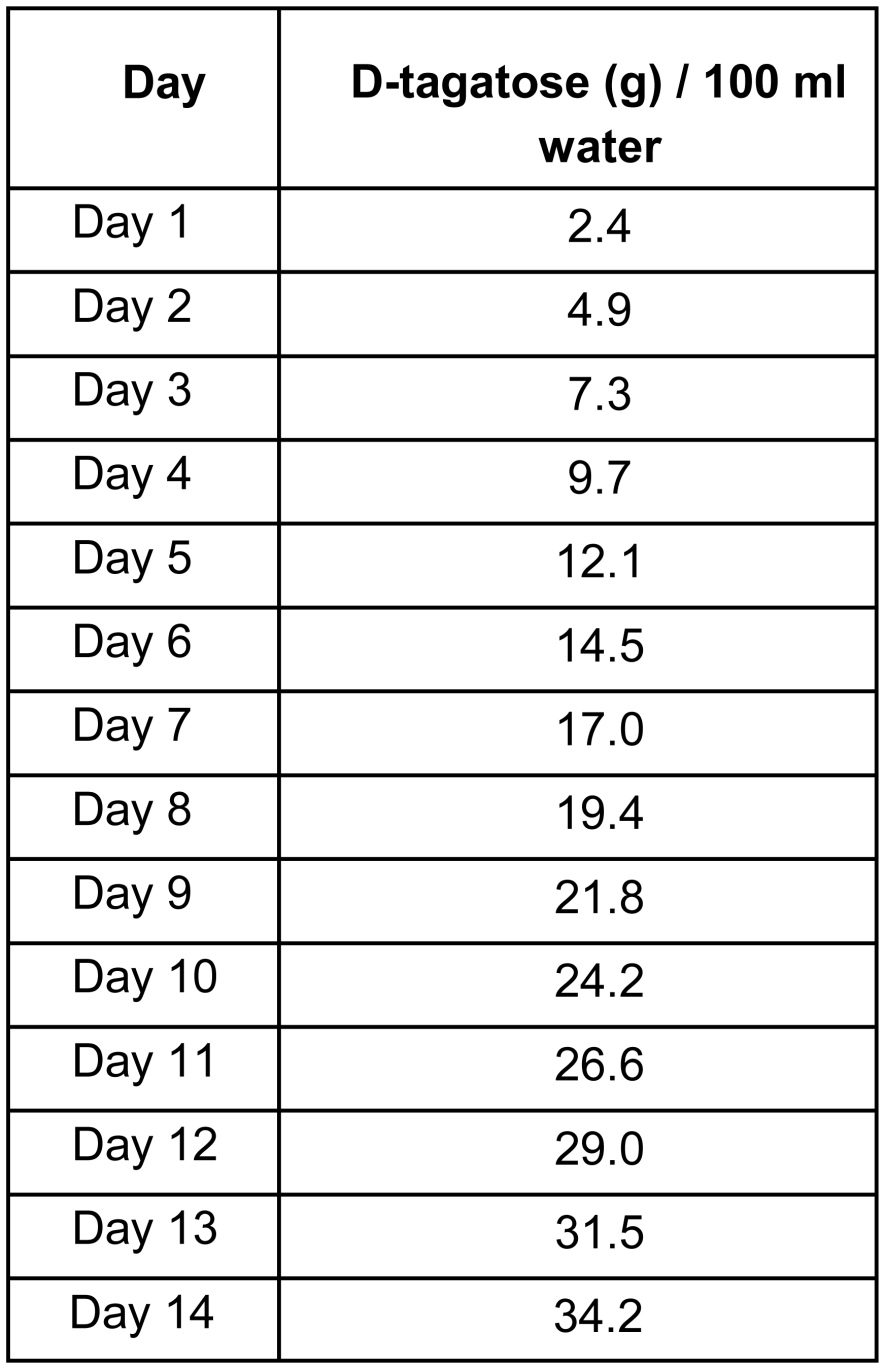
Addition of D-tagatose to drinking water during the two week run-in phase. Increasing amounts of D-tagatose were added to drinking water to acclimate mice to D-tagatose. Mice in Groups 3 and 5 had D-tagatose added to their drinking water.

**Illustration 3:**
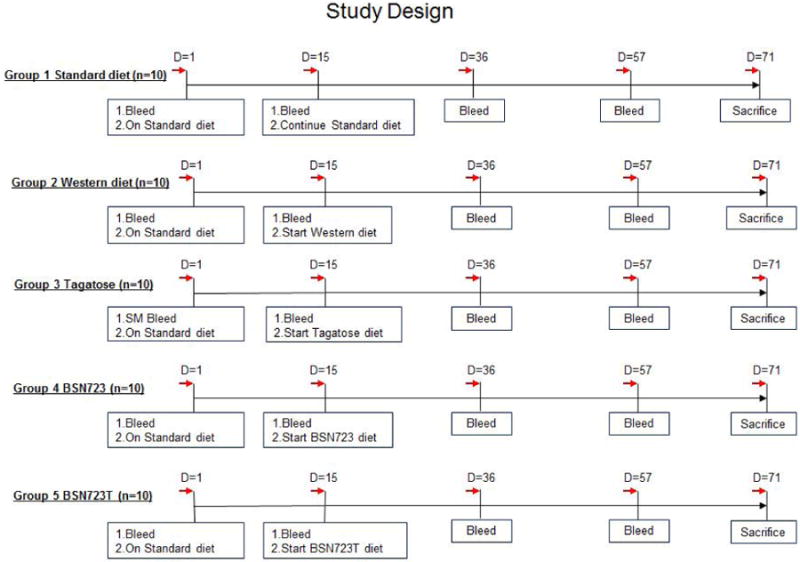
Outline of the study protocol. On day 0 mice were weighed and randomized to produce groups with the same mean body weight and fasted overnight. During the run-in period, mice in Groups 3 and 5 were given increasing amounts of D-tagatose in their water according to the schedule in Illustration 2.

**Illustration 4:**
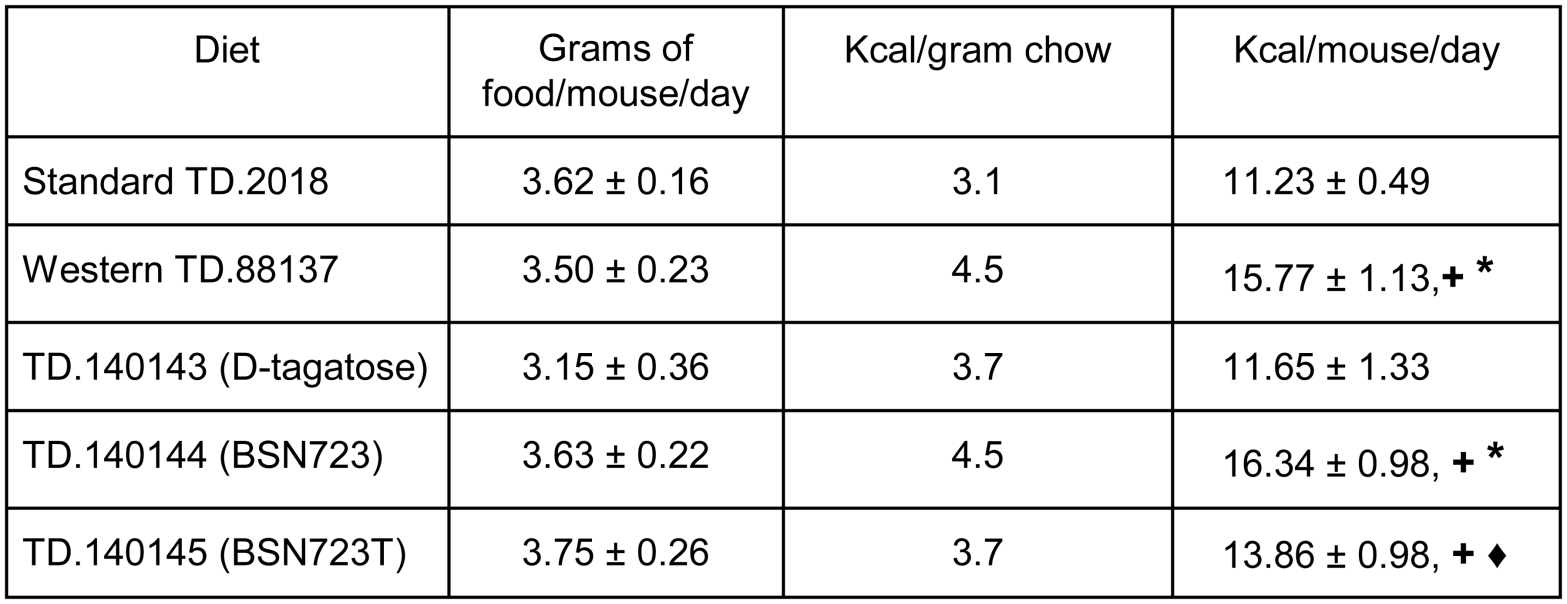
Comparison of food consumption and average caloric intake of mice according to diet. Values are reported as mean +/- s.e.m.

**Illustration 5:**
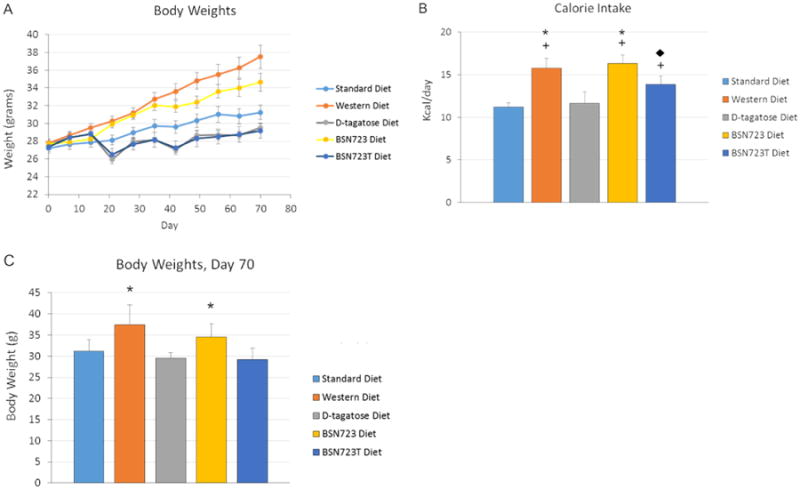
All mice were kept on the Standard diet during the two week D-tagatose run-in phase (days 0 to 14). Mice were then placed on their respective diets for eight weeks. (A) Change in body weights over time. (B and C) Caloric intake (B) compared to body weights (C) at the end of the study. Standard diet, n = 9-10; Western diet, n = 10; D-tagatose diet, n = 10; BSN723 diet, n = 9-10; BSN723T diet, n = 10. Results are reported as mean +/- s.e.m.

**Illustration 6:**
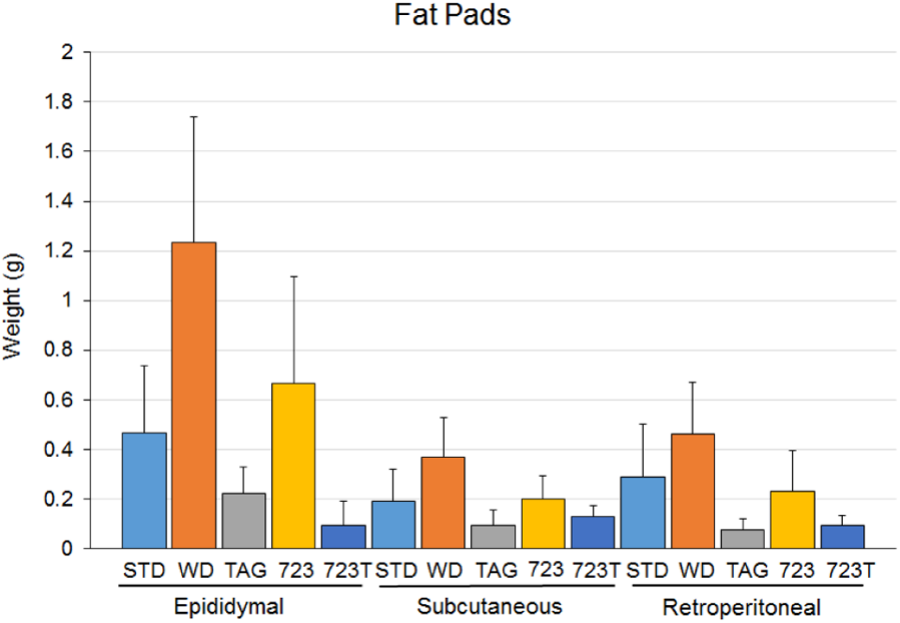
Adipose tissues from mice. Standard diet, n = 9; Western diet, n = 10; D-tagatose diet, n = 10; BSN723 diet, n = 9; BSN723T diet, n = 10. Results are reported as mean +/- s.e.m.

**Illustration 7:**
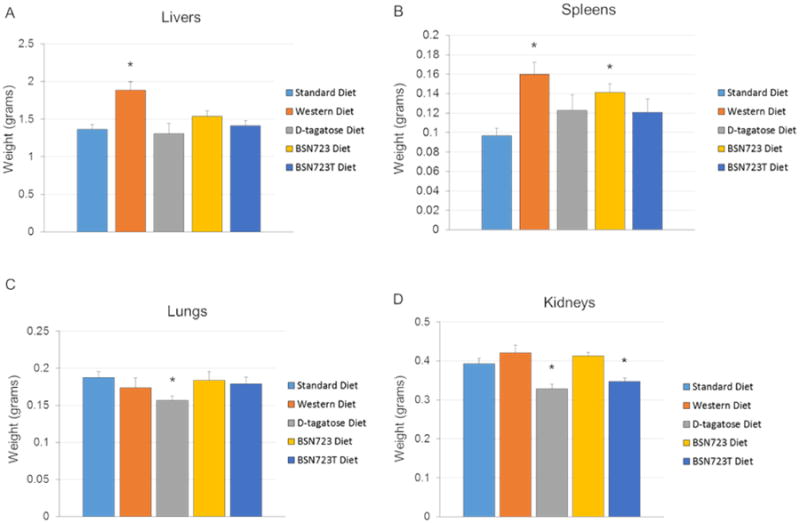
Organ weights. A) Livers. B) Spleens. C) Lungs. D) Kidneys. Standard diet, n = 9; Western diet, n = 10; D-tagatose diet, n = 10; BSN723 diet, n = 9; BSN723T diet, n = 10. Results are reported as mean +/- s.e.m.

**Illustration 8:**
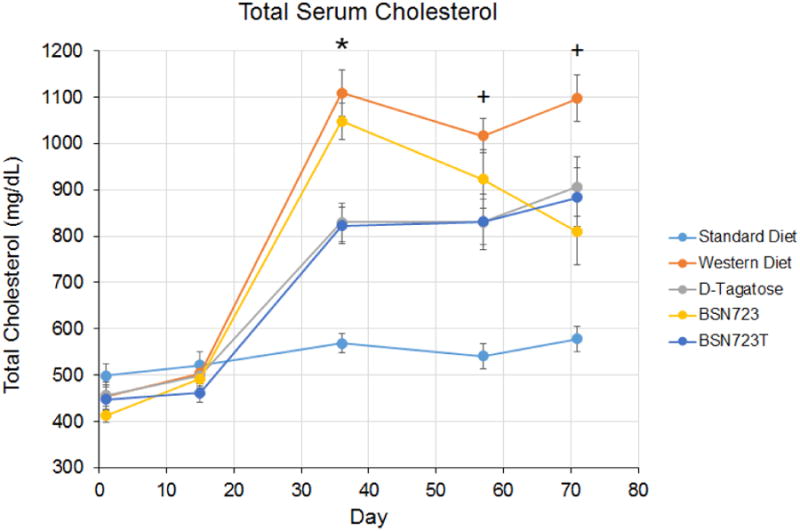
Time course of total serum cholesterol. All mice were fed Standard diet during the D-tagatose run-in (Days 1 to 14) and then placed on their respective diets. Standard diet, n = 9; Western diet, n = 10; D-tagatose diet, n = 10; BSN723 diet, n = 9; BSN723T diet, n = 10. Results are reported as mean +/- s.e.m.

**Illustration 9:**
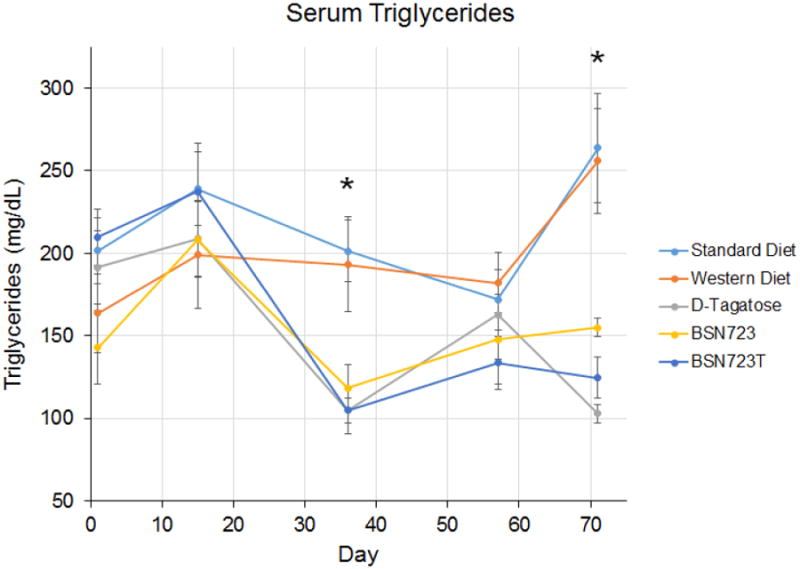
Serum triglycerides. All mice were on the Standard diet during the two week D-tagatose run-in period (Days 1 to 14) and then placed on their respective diets. Standard diet, n = 9; Western diet, n = 10; D-tagatose diet, n = 10; BSN723 diet, n = 9; BSN723T diet, n = 10. Results are reported as mean +/- s.e.m.

**Illustration 10:**
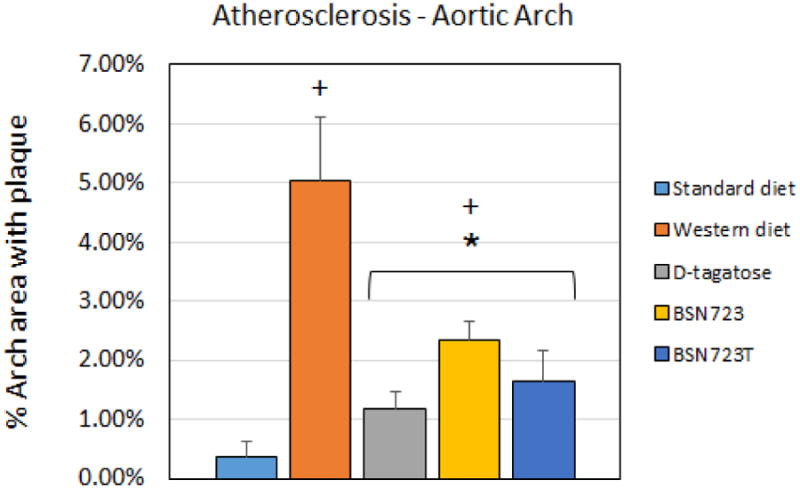
Comparison of the percent area of the aortic arch that has plaque. Aortas were prepared for atherosclerosis measurements via en face presentation. The addition of D-tagatose, dihydromyricetin or BSN723T to the Western diet significantly prevented the formation of atherosclerotic plaques. Standard diet, n = 9; Western diet, n = 10; D-tagatose diet, n = 10, BSN723, n = 9, BSN723T, n = 10. Results are shown as mean +/- s.e.m.

## Abbreviations

AAPH: 2,2′-azobis(2-methylpropionamidine) dihydrochloride
AMPK: phosphorylated AMP activated protein kinase
ApoE^-/-^: apolipoprotein E deficient
BSN723: dihydromyricetin
BSN723T: combination of dihydromyricetin and D-tagatose
DMY: dihydromyricetin
GLUT4: glucose transporter type 4
HbA1c: Glycated hemoglobin
HDL: high-density lipoprotein
IDL: intermediate density lipoprotein
LDL: low-density lipoprotein
LDLr^−/−^: low-density lipoprotein receptor deficient
MG: methylglyoxal
mTOR: mammalian target of rapamycin
PGC-1α: peroxisome proliferator-activated receptor γ coactivator 1α
s.e.m: standard error of the mean
SIRT3: sirtuin 3
ULK1: serine/threonine protein kinase
VLDL: very low density lipoprotein
